# Potential Therapeutic Application and Mechanism of Action of Stem Cell-Derived Extracellular Vesicles (EVs) in Systemic Lupus Erythematosus (SLE)

**DOI:** 10.3390/ijms25042444

**Published:** 2024-02-19

**Authors:** Sushmitha Rajeev Kumar, Rajalingham Sakthiswary, Yogeswaran Lokanathan

**Affiliations:** 1 Centre for Tissue Engineering and Regenerative Medicine, Faculty of Medicine, University Kebangsaaan Malaysia, Jalan Yaacob Latiff, Cheras, Kuala Lumpur 56000, Malaysia; sushmitharajeev97@gmail.com; 2 Department of Biomedical Engineering, Faculty of Engineering, University of Malaya, Lembah Pantai, Kuala Lumpur 50603, Malaysia; 3 Department of Medicine, Faculty of Medicine, University Kebangsaan Malaysia, Jalan Yaacob Latiff, Cheras, Kuala Lumpur 56000, Malaysia; sakthis5@hotmail.com

**Keywords:** extracellular vesicles (EVs), immunomodulation, inflammation, mesenchymal stromal cells, systemic lupus erythematosus (SLE), autoimmune

## Abstract

Systemic lupus erythematosus (SLE) is a multisystemic autoimmune disease that affects nearly 3.41 million people globally, with 90% of the cases affecting women of childbearing age. SLE is a complex disease due to the interplay of various immunological pathways and mechanisms. This scoping review aims to highlight the latest research findings on the therapeutic mechanisms of action of EVs in SLE. Relevant research articles were identified using the PRISMA framework from databases such as PubMed/MEDLINE (National Library of Medicine), Scopus (Elsevier), and Web of Science: Core Collection (Clarivate Analytics) from July 2023 to October 2023. Eleven studies met the inclusion criteria and thus were included in this scoping review. The findings showed that EVs have therapeutic effects on ameliorating the disease progression of SLE. EVs can reduce the pro-inflammatory cytokines and increase the anti-inflammatory cytokines. Moreover, EVs can increase the levels of regulatory T cells, thus reducing inflammation. EVs also have the potential to regulate B cells to alleviate SLE and reduce its adverse effects. The scoping review has successfully analysed the therapeutic potential in ameliorating the disease progression of SLE. The review also includes prospects to improve the effects of EVs further to increase the therapeutic effects on SLE.

## 1. Introduction

Systemic lupus erythematosus (SLE) is a multisystemic autoimmune disease with an increased risk of morbidity and mortality [1,2]. This autoimmune disease is detected in women between adolescence and climacteric ages [3]. Like most autoimmune diseases, the cause of SLE development is still undetermined. However, genetic and environmental conditions can influence the pathogenesis of SLE [4]. Importantly, diagnosing this disease at an earlier stage helps reduce the disease progression and organ damage [5]. Late diagnosis of SLE and inadequate treatment can lead to uncontrolled chronic inflammation and multisystemic complications, including maculopathy, transaminitis, allergies, cytopenia, and joint deformities [6,7,8,9]. This can lead to a poor quality of life, affecting the productivity of both work-related and nonwork-related activities [10]. This can result in a direct effect on the patient’s mental health, leading to depression and anxiety [11].

**Figure 1:**
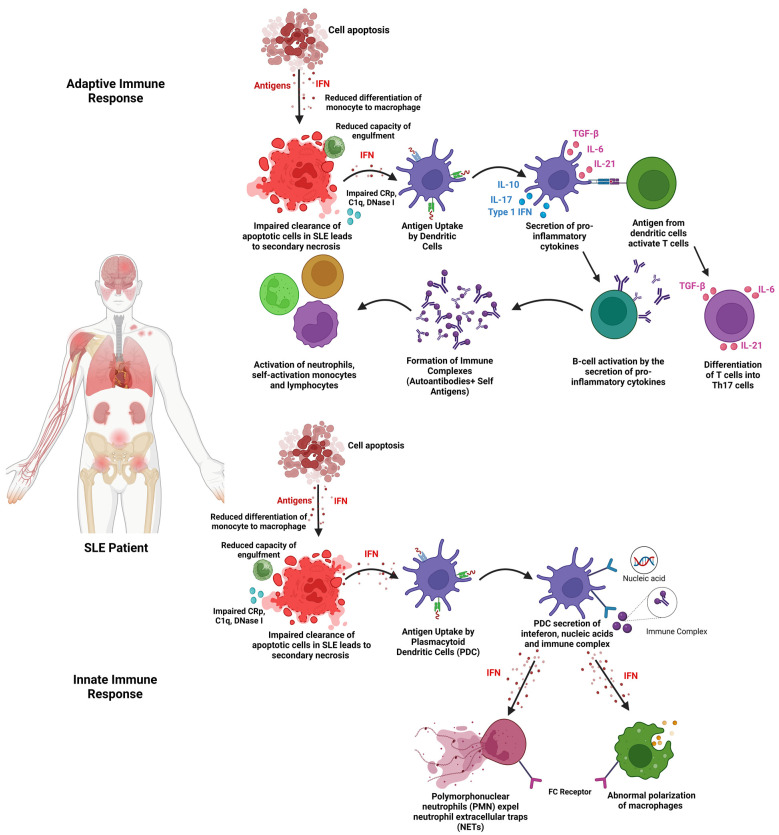
Pathogenesis of the innate and adaptive immune response in SLE.

SLE involves both innate and adaptive immune responses with the overt production of immune complexes and autoantibodies. SLE can also be described as the loss of immunological resistance against self-antigens, resulting in the formation of autoantibodies involved in disease pathogenicity, causing tissue damage through various immunopathogenic pathways [2]. Dysregulation of apoptotic cell clearance affects both the innate and adaptive immune responses (Figure 1). In the innate immune response, impaired clearance of apoptotic cells dysregulates the type 1 interferon [12]. This dysregulation of the type 1 interferons increases the expression of neutrophil extracellular traps (NETs) from polymorphonuclear cells (PMN). This induces the secretion of pro-inflammatory cytokines [13]. The dysregulation of type 1 interferons also influences macrophage polarisation. Impaired clearance of apoptotic cells triggers the adaptive immune response via the overactivation of T cells and B cells [13]. The overactivation of T cells and B cells increases the stimulation of neutrophils, self-reactive lymphocytes, and monocytes [14]. The increase in immune cells increases the secretion of pro-inflammatory cytokines, thus developing an autoimmune reaction involved in the disease pathogenesis of SLE.

As many organs and tissues are affected by SLE, this autoimmune disease is heterogeneous, with the clinical representations evolving with time [12]. The treatment of SLE mainly involves the use of immunosuppressants to control chronic inflammation and prevent organ damage [15,16]. However, these therapies are associated with adverse effects such as cancer, osteoporosis, diabetes induced by steroids, and avascular necrosis at joints [15]. Many patients have refractory disease despite standard therapies. Thus, there is an ongoing search for new agents and methods to achieve disease remission.

## 2. Extracellular Vesicles (EVs)

EVs have surfaced as a therapeutic agent in immunotherapy, regenerative medicine, and tissue engineering due to their characteristics that promote immunomodulatory properties and the potential to induce tissue regeneration [17,18]. Moreover, EVs are highly biocompatible due to their low levels of immunogenicity and toxicity when used for therapeutic purposes [19]. EVs consist of exosomes, apoptotic vesicles, and microparticles that are released by cells, categorised based on the size range in diameters of (~40 nm–160 nm), (500 nm–2 µm), and (100 nm–500 nm), respectively [18,20]. EVs are usually derived from body fluids such as urine, amniotic fluid, and blood [21]. A variety of cells ranging from macrophages, dendritic cells (DCs), mesenchymal stem cells (MSCs), epithelial cells, platelets, lymphocytes, and fibroblasts secrete EVs. EVs must be isolated and characterised to understand the size, shape, density, surface charge, and porosity, which have a direct effect on biological interactions. Common characterisation methods include flow cytometry, nanoparticle tracking analysis (NTA), transmission electron microscopy (TEM), resistive pulse sensing (RPS), and atomic force microscopy (AFM) [22]. Different cells secrete EVs carrying different proteins and messages of the cells. [23]. For example, EVs derived from macrophages will carry inflammatory cytokines that are secreted by the macrophages, which can either produce an anti-inflammatory effect or a pro-inflammatory effect [23,24]. Meanwhile, EVs derived from MSCs can induce tissue regeneration, promote various differentiation pathways, and regulation of the immune system [25].

EVs are released in various biological processes in the body that can be observed during cell motility, proliferation, apoptosis, differentiation, and immune response [26,27,28,29]. The correlation between the release of EVs during this process led to possible clinical approaches in disease pathogenesis and treatment of diseases. EVs are responsible for intercellular communications between cells [30]. The functionality of EVs depends entirely on the intercellular communication between cells and EVs [31]. This pathway can be targeted as a potential treatment mechanism in diseases such as cancer [32], neurological diseases [33] and metabolic-related diseases [34]. Apart from intercellular communication, EVs have surfaced as biomarkers in the diagnosis and management of the progression of the disease [18]. The biomarkers carry proteins, metabolites, and nucleic acid that provide information on the disease [35]. The nanosize of EVs highlights their potential in delivering drugs not only to targeted tissues but also helps in crossing the blood–brain barrier to target neurological conditions [36]. These excellent characteristics of EVs further prove their diagnostic and therapeutic potential in complex diseases.

EV research has shown an upward trend in the last 5 years, proving the therapeutic potential of EVs in ameliorating various spectrum of diseases. In various studies, stem-cell-derived EVs have shown notable progress in the therapy of cancer. In a study by Zhu et al. [37], NK cell-derived EVs were able to suppress tumour progression and increase cytolytic levels in human cancer cell lines. A study by Bruno et al. [38] proved that EVs derived from BM-MSCs could inhibit tumour progression significantly, thus ameliorating the progression of the disease. Apart from cancer, therapeutic effects of EVs were observed in neurological disorders as well. EVs derived from MSCs of human adipose tissue have a positive effect on Alzheimer’s disease. The secreted EVs carry enzymatically active neprilysin that clears the accumulation of amyloid-β (Aβ) in Alzheimer’s disease [39]. Apart from Alzheimer’s, a study by d’Angelo et al. [40] recorded the potential of MSC-derived secretome as therapy for Parkinson’s disease. The secretomes could prevent neuroinflammation and enhance the neurotrophic factor expression that improves the check on the progression of Parkinson’s disease.

The therapeutic effects of EVs in other complex diseases have paved the way as a potential treatment for autoimmune diseases. The therapeutic potential can be observed in many studies, such as rheumatoid arthritis and type 1 diabetes. In rheumatoid arthritis (RA), in vivo studies have reported immunosuppressive properties of EVs that inhibit the proliferation of T lymphocytes and reduction in pro-inflammatory cytokines IL-6, TNF-α, and IL-1β [41]. Moreover, EVs have shown significant positive effects in studies involving type 1 diabetes by enhancing the secretion of anti-inflammatory cytokines while decreasing the levels of pro-inflammatory cytokines [42]. These findings suggest that EVs have the potential therapeutic effects in preventing and reducing the disease progression of autoimmune disorders, including SLE. The interest in the therapeutic effects of EVs in SLE has led to this scoping review. This scoping review aims to highlight the latest research findings and the therapeutic mechanism of action of EVs on SLE while identifying research gaps for future research. Besides discussing the application of EVs specifically on SLE and its mechanism in detail, the paper also discussed the role of miRNAs and tsRNAs in EVs in alleviating SLE and the mechanism of action. In addition, the dosage of EVs and animal models used were summarised.

## 3. Methods

The scoping review was based on research literature obtained using the Preferred Reporting Items for Systematic Reviews and Meta-Analyses PRISMA framework (Figure 1). The research literature obtained was reviewed and validated by other authors of this paper.

## 4. Identification of Relevant Studies

The inclusion criteria used in this scoping review were based on the following: (a) original research papers, (b) papers including in vivo, ex vivo, or in vitro model of study, (c) therapeutics for SLE involving EVs, (d) papers from the year 2013 to 2023, and (e) papers only written in English. Sources were excluded if they were not original research studies, including reviews, opinions, articles, books, and conference papers. Studies involving plant-derived EVs were excluded. Apart from that, EVs, including exosomes, apoptotic bodies, and microparticles, are included. Studies that relate to the definition of EVs, such as “lipid-bound vesicles secreted by cells into the extracellular space with the size range of (30 nm–10 µm)”, are included.

To identify the relevant studies based on the title, the literature searches were carried out on the following databases: PubMed/MEDLINE (National Library of Medicine), Scopus (Elsevier), and Web of Science: Core Collection (Clarivate Analytics). The literature search was carried out from 29 July 2023 to 16 October 2023. Filters were applied during the literature search to identify English articles published between 2013 and 2023. Apart from that, additional papers relevant to this review article that were not found through the databases were manually searched.

A specific combination of keywords and synonym vocabulary terms were used to identify the research papers in the searched database. For the PubMed/Medline database, Medical Subject Headings (MeSH) were used as a guideline to identify relevant keywords based on the title of the review paper. The same keywords were used in other database searches as well. The keywords and their synonyms were used to describe every part of the review article. The terms included were exosome, extracellular vesicle, vesicle, EV, systemic lupus erythematosus (SLE), lupus erythematosus, treatments, therapeutics, and interventions. The * sign was used to include plural keywords in the search databases. The Boolean operators ‘AND’ and ‘OR’ were used in the database search. ‘AND’ includes all the keywords stated, while ‘OR’ includes either one of the keywords. The search terms that were used to retrieve the articles were as follows: (“exosome*” OR “extracellular vesicle*” OR “vesicles” OR “EVs”) AND (“lupus erythematosus” OR “systemic lupus erythematosus”) AND (“TREATMENT*” OR “THERAPEUTIC*” OR “INTERVENTION”).

The database search was limited to English language articles from 2013 to 2023. Article type was not limited during the database search.

## 5. Study Selection

The research papers obtained from the database search were compiled in an EndNote library (Clarivate Analytics, version X21). The duplicate records were removed, and the other research articles were reviewed based on their title and abstracts for eligibility. The full-text articles were then reviewed for their eligibility. The exclusion and inclusion criteria were taken into consideration during the screening process.

## 6. Collecting, Summarising, and Reporting Results

The contents and data extracted were analysed and presented in a table for a clearer view. The table includes the EVs/miRNA studied, the disease model, the source of EVs, the characterisation method, related modifications, and the disease outcome. The data were examined based on the topics for similarities and differences. The therapeutic mechanism of action of EVs on SLE and the research trends were summarised.

## 7. Results

**Figure 2:**
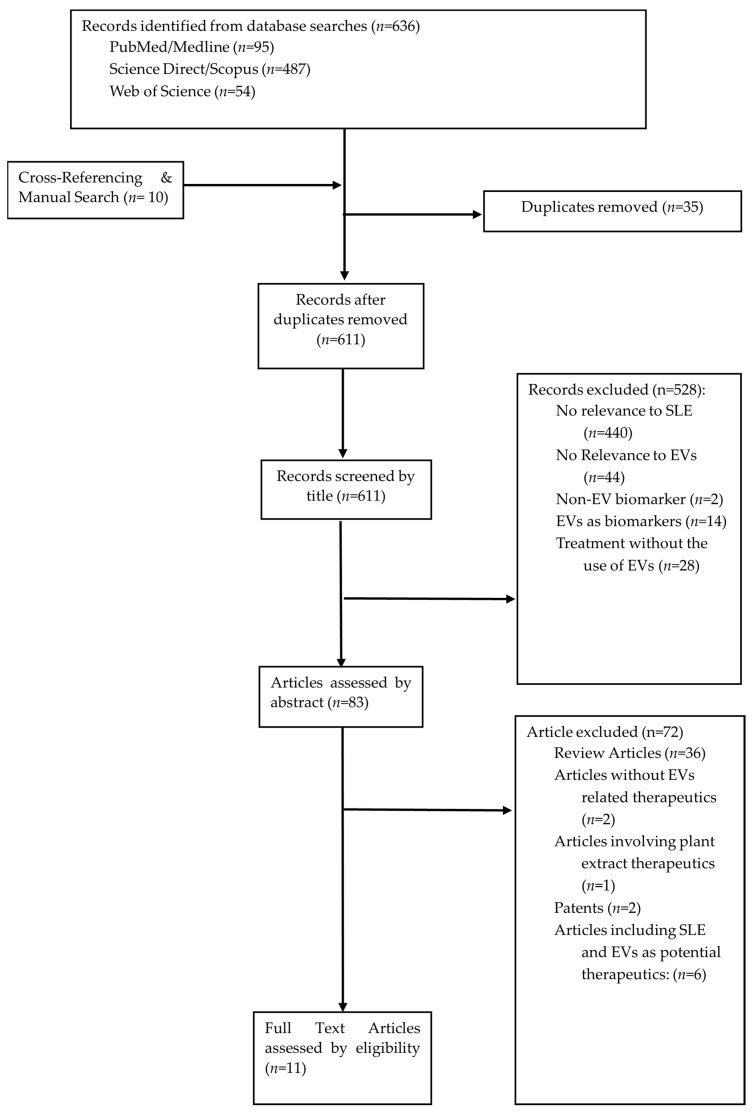
Preferred Reporting Items for Systematic Reviews and Meta-Analyses (PRISMA) Protocol.

The database searches identified a total of 636 publications, including articles that were manually searched, which are recorded in Figure 2. A total of 35 duplicates were removed, and a total of 611 articles were screened based on the titles. In total, 528 records were excluded, with 440 articles excluded due to their irrelevance to SLE. A total of 14 papers were excluded as EVs were identified as biomarkers instead of therapeutics. A total of 44 papers were excluded due to irrelevance to EVs. Two articles were excluded as the paper mentioned non-EV biomarkers. Moreover, 28 articles were excluded as the treatment method did not involve EV vesicles. Eighty-three articles were then screened based on the abstracts, and a total of seventy-two articles were excluded, with thirty-four articles being review articles, two articles without the use of EVs, and one article based on plant-derived EVs. Two searches were excluded as they were patents, and a total of six had mentioned the future potential of EVs as therapeutics for SLE. A total of 11 full-text articles were assessed for their eligibility, and all 11 articles were included in this review (Figure 2). A total of nine studies mentioned exosomes as the type of EVs used, while one study mentioned apoptotic vesicles, and another study mentioned EVs.

## 8. Discussion

### 8.1. Isolation of EVs

The studies gathered in this review include isolation methods of EVs from mesenchymal stem cells of various origins, such as bone marrow, umbilical cord, umbilical cord blood, adipose tissue, and deciduous tooth pulp. The isolation method of EVs is crucial to obtain the possible highest purity of EVs to further enhance the specific mechanism of action required [22]. In all the studies, EVs were isolated from the supernatant only after reaching 80–90% confluency of MSCs. The supernatant derived from the conditioned media undergoes centrifugation between 10,000× *g* and 125,000× *g* and further undergoes ultracentrifugation at 140,000× *g* to isolate the EVs based on the size from the precipitate obtained [43]. Apart from that, some studies have included EVs that are also isolated from supernatants using super high-speed centrifugation of 175,000× *g*. There are other studies that have stated the use of only ultracentrifugation between 125,000× *g* and 140,000× *g* for the isolation of EVs [1,44,45,46,47]. Some studies have isolated EVs from respective MSCs using isolation kits. One study indicated that EVs from adipose-derived mesenchymal stem cells (ADSCs) were isolated using an isolation kit (ExoQuick-TC)-System Bioscience, Palo Alto, CA, USA, where the isolation reagent was added into the cell pellet overnight and centrifuged to obtain the EVs [48]. Another study indicated that the EVs were isolated from a Cell Culture Media Exosome Purification Mini Kit-Norgen Biotek, Thorold, ON, Canada [49]. Moreover, the isolation of EVs from deciduous tooth pulp stem cells (SHED) was carried out from cultured media using an exoEasy Maxi Kit-Qiagen, Valencia, CA, USA [50]. In a study involving BM-MSCs, the EVs were isolated from conditioned media using the size-exclusion chromatography (qEV) exosomes isolation kit-iZON Science, Cambridge, MA, USA [51]. The study by Wang et al. [52] did not include isolation methods of apoptotic vesicles (ApoVs) derived from BM-MSCs; however, the study included a method of inducing apoptosis in BM-MSCs using staurosporine (STS) to secrete ApoVs.

### 8.2. Characterisation of EVs

The isolated EVs are required to be characterised based on their size and specific EV markers to identify the protein expressed by the EVs [43]. From the studies, the common characterisation method of EVs is the Western blot assay (Table 1). The Western blot assay is used to determine specific protein markers related to EVs, which are commonly identified as CD9, CD36, and endoplasmic reticulum-oriented calnexin [43]. Other protein markers found in the selected studies that were used to identify and characterise EVs include CD63, Alix, CD81, CD63, GAPDH, and TSG101 [43,44,45,46,47,48,49,50,51]. Characterisation of EVs based on morphology was conducted using a transmission electron microscope and was recorded in a few of the studies. The morphology of EVs that were reported in the studies includes EVs shaped like a saucer, round, or sphere-shaped vesicles that include an entire capsule, bilayer-membrane structure, and a hollow globular vesicle [1,43,44,45,49,51]. Apart from that, most studies included the characterisation of EVs by size, using either a nanoparticle tracking analyser or a particle tracking assay [1,43,44,45,50]. However, some studies included a different characterisation method to understand the uptake of EVs using the ExoGlow-Protein EV labelling kit-(Green, System Bioscience, Palo Alto, CA, USA) that dyes EVs fluorescent green [51]. The study involving SHED-EVs reported a unique characterisation method based on the Ag expression on the surface of the EVs that were analysed using ExoAB Ab kit (System Bioscience, Palo Alto, CA) and R-PE-conjugated anti-rabbit IgG Ab (Cell Signalling Technology, Dancers, MA) using flow cytometry (BD Biosciences) [50]. One study also mentioned the characterisation method to determine the diameter of EVs derived from human umbilical cord mesenchymal stem cells (hUC-MSCs) using a dynamic activation scattering analysis [47]. Apart from characterising EVs, the study by Wang et al. [52] characterised apoptotic vesicles (ApoVs) using an apoptosis marker, PtdSer/PS, to identify the ApoVs secreted by the BM-MSCs. These characterisation methods in the papers are in line with the guidelines by (Minimal Information for Studies of Extracellular Vesicles) MISEV. The guidelines include the characterisation of EVs based on topology, where the protein or RNA present in the EVs are identified for characterisation [53].

### 8.3. Range of Dose of EVs Administered

To investigate the efficacy of EV treatment for SLE in in vivo models, the dose of EVs required is an important factor that can determine the immunomodulation effects in SLE. In the study of Chen et al. [43] and Chen et al. [46], the mice were injected with 100 μL of 0.2 mg/mL EVs derived from hUC-MSCs via intravenous injection through the tail vein every 2 days for 14 days. Instead of 2 days, the study carried out by Wei et al. [48] reported 2 × 10^5^ cells per 10 g animal weight of ADSC/miR-20a per 150 μL PBS solution weekly for 14 days. Instead of multiple injections of EVs, a study by Sun et al. [47] recorded single doses of EVs of 200 μg based on the protein concentration that were administered. Apart from that, the study by Sonoda et al. [50] reported the administration of 100 μg of SHED EVs in 100 mL of PBS solution once every 4 weeks. Moreover, the study by Wang et al. [52] on ApoVs only included the frequency of administration, which is weekly for 4 weeks. All the range of doses of EVs administered is recorded in Table 2. Other studies were excluded in this section of the discussion due to no information provided on the dose of EVs administered in the in vivo studies.

### 8.4. Mechanism of Action (EVs)

#### 8.4.1. Effects of EVs on Pro-Inflammatory Cytokines

The immunomodulatory characteristics of EVs have championed the role of EVs in downregulating disease progression of SLE through various pathways and mechanisms. In terms of macrophage polarisation, in the study by Chen et al. [43], EVs derived from hUC-MSCs are involved in decreasing the levels of NOTCH1, IL-1β, and iNOS which are markers indicating activation of the M1 phenotype (pro-inflammatory). Specifically, the iNOS marker was also downregulated when BM-MSC EVs were used [51]. The effect of EVs on macrophage polarisation is also proven in the study by Dou et al. [49], where EVs derived from human bone marrow mesenchymal stem cells (BM-MSCs) decreased the polarisation of macrophage into the M1 phenotype. The expression of CD80, NOS2, and MCP-1, which are protein expression markers in M1 macrophages, was significantly decreased. Moreover, a study by Sun et al. [47] recorded different markers, such as CD14+ and CD11c+, that indicate M1 macrophage polarisation.

The EVs also had a positive effect on the downregulation of the secretion of pro-inflammatory cytokines TNF-α, IL-6, IL-16 IL-12, IFN-γ, IL-1β, IL-17, and GM-CSF, indicating depolarisation of macrophages into the M1 phenotype [1,43,44,45,46,47,48,49,50,51]. However, in the study by Zhao et al. [45], IL-6 (pro-inflammatory cytokine) levels showed signs of upregulation due to the possibility of paracrine effects of MSCs that elevate IL-6 levels.

#### 8.4.2. Effects of EVs on Anti-Inflammatory Cytokines

Moreover, the EVs played a pivotal role in increasing the CD206, CD86+, CD116+, Arginase-1, and IL-10, which are part of markers indicating M2 macrophage polarisation leading to anti-inflammatory effects [43,46]. CD206+ and CD163+ markers can also be seen to be upregulated in the study carried out by Sun et al. [47] using UC-MSCs to inhibit lupus via M2 macrophage polarisation. The upregulation of Arg-1 markers is also recorded in the study carried out by Zhang et al. [51] involving EVs derived from BM-MSCs. The study by Dou et al. [49] also recorded an upregulation of CD206, MRC-2, and ARG-1, which are M2 macrophage markers indicating an anti-inflammatory response. Different markers of M2 macrophage, such as CD14+ and CD163+, were alleviated and recorded in the study by Sun et al. [47]. In a study by Zhang et al. [51], the levels of reactive oxygen species were observed, and EVs have successfully reduced ROS levels that indicate the polarisation of macrophages into the M2 phenotype (anti-inflammatory).

The EVs also alleviated the levels of anti-inflammatory cytokines IL-10, TGF-β, and M-CSF. The secretion of anti-inflammatory cytokines indicates the polarisation of macrophages into the M2 phenotype. [1,43,44,45,46,47,48,49,50,51].

#### 8.4.3. Effects of EVs on T Cell Lineage

Apart from the secretion of anti-inflammatory and pro-inflammatory cytokines, EVs have proven to be involved in the regulation of Treg and T helper cells to suppress the disease progression of SLE. In the study by Tu et al. [44], Th17 subsets were significantly downregulated, showing a reduction in pro-inflammation effects. The cytokine level IL-17 co-relates to the production of Th17 cells. Lower levels of IL-17 indicate lower production of Th17 cells, as IL-17 acts as a biomarker to examine the disease activity in SLE patients [54]. Although SLE pathogenesis involves higher differentiation of Th17 cells and increased levels of IL-17 cytokines, the study by Xie et al. [1] recorded an opposite effect. In that study, hUC-MSC-EVs promoted the differentiation of Th17 cells and increased IL-17 cytokines while inhibiting CD4+ T cells, hypothesising that increased levels of IL-17 and Th17 promote immune-regulatory effects. In terms of Treg cells, the study by Sonoda et al. [50] and Sun et al. [47] recorded an upregulation of CD4+, CD25+, and FoxP3+ biomarkers indicating inhibition of T cell activation. A study carried out by Zhang et al. [51] also proved EV potential in immunomodulation by increasing the differentiation of T cells into Treg cells. Apoptotic vesicles (ApoVs) have been shown to have positive effects on T cells. The study by Wang et al. [52] increased the CD4+ T effector cells while decreasing the CD4+ naïve T cells. Moreover, ApoVs reduced the expansion of Th1, Th17, and Th2, which are T cell subsets that downregulated pro-inflammatory cytokines levels of IFNγ+, CD4+, IL-17A+, and IL-4.

#### 8.4.4. Effects of EVs on B Cells

Having said that, EVs also have the potential to regulate B cells, which was recorded in the study carried out by Zhao et al. [45], which further confirms immunomodulation effects via B cells. EVs have been shown to increase the levels of B cell apoptosis while inhibiting the excessive proliferation of B cells. Cytokine levels further confirmed that the hyperactivation of B cells reduced significantly after the treatment with EVs.

#### 8.4.5. Effects of EVs on Lupus Nephritis (c-Complements)

Furthermore, SLE can also lead to complications such as lupus nephritis, which is caused by kidney inflammation. EVs were also reported in various studies, proving to have positive effects by alleviating inflammation and its effects on the kidneys. In the study by Wei et al. [48] and Zhang et al. [51], levels of IgG and C3, which are immune complexes, were significantly downregulated in the glomerular mesangial and endocapillary of the kidney after the treatment with EVs. Furthermore, other immune complexes such as IgM and C1q were significantly reduced in studies recorded by [51]. EVs can also downregulate levels of dsDNA, serum creatine in blood, and protein levels in urine produced by patients with SLE [48]. With regard to lupus nephritis, a study by Mohd et al. [55] recorded the co-relation of gut microbiota to lupus nephritis in mediating the immune complex deposition and macrophage infiltration in the renal system. Targeting EVs to improve the gut microbiota proves to be a potential treatment for lupus nephritis. The study by Luo et al. [56] proved the theory of the potential of EV-derived miRNAs obtained from the intestinal tract to improve the homeostasis of the gut to inhibit the disease progression of SLE. This indication further confirms the therapeutic effects of EVs in SLE pathogenesis, precisely in lupus nephritis.

### 8.5. Role of miRNAs and tsRNAs in Extracellular Vesicles in Ameliorating the Disease Progression of SLE

**Figure 3:**
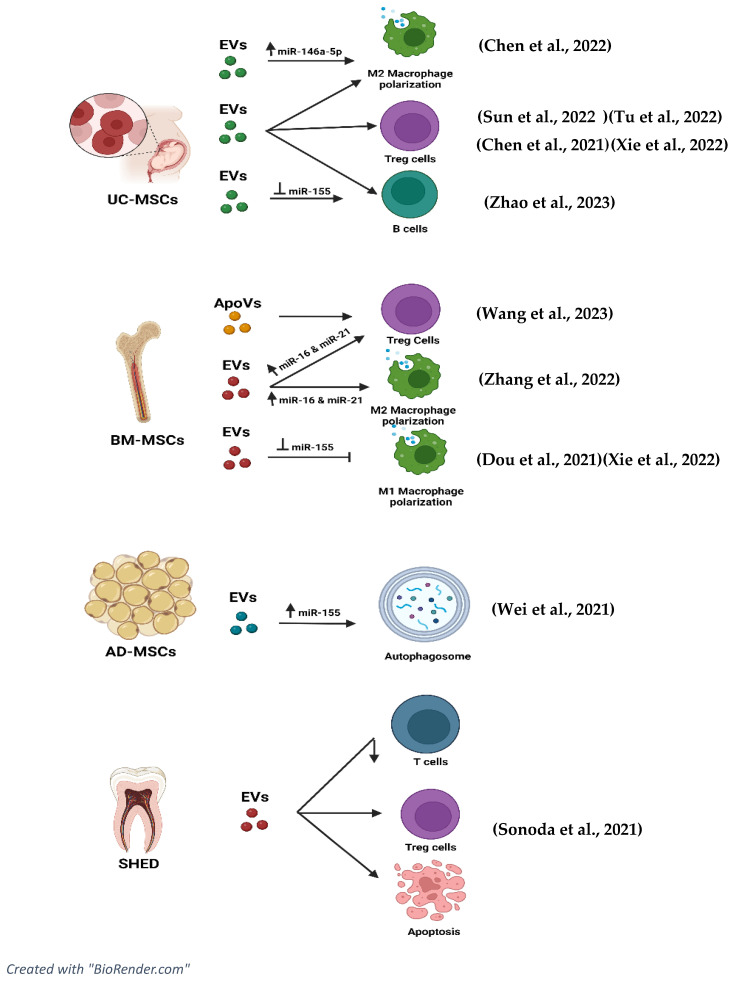
EVs and miRNAs that are related in ameliorating the disease progression of SLE. BMMSCS, bone marrow mesenchymal stromal cells; SHED, stem cells from human exfoliated deciduous teeth; hucMSCs, human umbilical cord mesenchymal stromal cells; AD-MSCS, adipose-derived mesenchymal stromal cells; UC-MSCs, umbilical cord mesenchymal stromal cells [1,43,44,45,46,47,48,49,50,51,52].

To further improve or understand the immunomodulatory effects of EVs, the effectiveness of inhibition and overexpression of miRNAs related to EVs are included in the studies and shown in Figure 3. The study by Chen et al. [43] indicated that the inhibition of *miR-146a-5p* resulted in adverse effects on lung injuries. NOTCH1, IL-1β, and iNOS were overexpressed while IL-10 and TGF-β levels were downregulated. The inhibition produced a negative effect, thus proving that expression of *miR-146a-5p* in EVs can upregulate IL-10, CD206, Arg-1, and TGF-β and decrease the expression of NOTCH1, IL-1β, and iNOS in patients diagnosed with SLE.

Another study by Tu et al. [44] investigated the expression of *miR-19b* in EVs derived from UCB-MSCs to further express *miR-19b* in T cells. The results indicated that miR-19b expressed in T cells via EVs lowered *TNF*, *IL-6*, and *IL-17* expression levels and increased *IL-10* and *TGF-beta* levels. The *miR-19b* also inhibited the expression of *KLF13* in T cells. The study also indicated that EVs could promote the T cells to express more *mir-19b,* which can inhibit the levels of *KLF3* that regulate the balance of Th17/Treg cells. The *miR-19b* also increases the production of anti-inflammatory cytokines *IL-10* and *TGF-beta* while downregulating levels of *IL-16*, *IL-17*, and *TNF-alpha*.

With regard to miRNA, another study by Wei et al. [48] recorded the isolation of *miR-20a* from EVs derived from ADSCs and the overexpression of *miR-20a*. The overexpression of *miR-20a* has recorded a positive effect on lupus nephritis models where anti-dsDNA antibody, urine protein, and serum creatinine levels were significantly lowered. It also triggered higher autophagy markers of Beclin 1, LC3-II/LC3-I, and p62 that indicated higher levels of autophagosomes. The autophagy mechanism stimulated the reduction in podocyte damage and reduced histopathologic abnormalities in the kidney.

A similar study carried out by Zhang et al. [51] investigated the effects of overexpression of miR-16 and *miR-21*. The study recorded decreased levels of iNOS, PDCD4, and PTEN. It also recorded lower pro-inflammatory cytokines and M1 macrophage markers that can be observed from the lower expression of CD86 and iNOS. The anti-inflammatory markers can be observed from the increased expression of CD206, Arg-1, B7H4, and CD138. The delivery of EVs through *miR-16* and *miR-21* targets PDCD4 and PTEN by higher polarisation of macrophages. The EVs expressing higher *miR-16* and *miR-21* showed higher efferocytosis of apoptotic cells and higher production of Treg cells (IL-17+ and Foxp3+).

Apart from miR-16 and miR-21, the study by Zhao et al. [45] indicated that the inhibition of *miR-155* in EVs can increase levels of B cell apoptosis while reducing B cell activation and proliferation. Inhibition of miR-155 can downregulate the production of inflammatory cytokines IL-6 and TNF- α, indicating immune regulating properties that can inhibit the disease progression of SLE.

Apart from miRNA, inhibition of *tsRNA-21109* in the study by Dou et al. [49] led to the increase in TNF-α and IL-1β, CD80, NOS2, and MCP-1 markers and a decrease in M2 markers CD206, MRC-2, and ARG-1. This indicated that the expression of tsRNA-21109 can inhibit M1 macrophage polarisation and induce M2 macrophage polarisation. Moreover, a study by Sonoda et al. [50] included *siRNATert* to understand the TERT expression and its effect on immune regulation. miR346 that binds to a region of 39-UTR of the TERTmRNA upregulates TERT expression and improves the TERT telomerase activity pathway while showing improvement in immune regulation.

### 8.6. EVs and Signalling Pathways

It is important to understand the effects of EVs and their specific miRNA and tsRNA on the pathway that is involved in the disease progression of SLE (as shown in Supplementary File S1). This is to ensure further understanding of the mechanism of action of EVs in immunomodulation. The pathway involved in the disease progression of SLE can be referred to in Figure 3. In the study by Chen et al. [43], the NOTCH 1 pathway was identified to play a crucial role in macrophage polarisation into the M1 phenotype. In this study, miR-146a-5p was found to inhibit the NOTCH 1 pathway, leading to M2 macrophage polarisation. Further reduction in pro-inflammatory cytokines can be observed when the NOTCH 1 pathway is targeted by *miR-146a-5p*.

Apart from the NOTCH 1 pathway, inhibition of the MAPK/ERK signalling pathway plays an essential role in regulating B cells and inhibiting B cell overactivation in SLE patients. The SHIP-1 protein levels have a direct effect on the ERK signalling pathway. To prove the mechanism of action of EVs through this pathway, *miR-155* was inhibited in B cells through EVs. The inhibition of miR-155 increased the expression of SHIP-1 proteins. Increased levels of SHIP-1 protein inhibit the ERK signalling pathway, thus reducing B cell proliferation and activation while increasing B cell apoptosis [45]. In a similar study by Dou et al. [49], the expression of *tsRNA21109* inhibited M1 macrophage polarisation through the signalling pathway of Rap1, Ras, Hippo, Wnt, MAPK, and TGF-beta which are inflammatory-related pathways involving macrophage polarisation.

Other inflammatory-related pathways that were included in the studies include the T cell receptor signalling pathway. The TCR pathway is involved in the regulation of cytokines, survival of T cells, proliferation, and differentiation of T cells. Dysregulation of this pathway can increase the chances of developing SLE [57]. The study by Wang et al. [52] recorded the decreased phosphorylation of the TCR signalling pathway when ApoVs were used in ameliorating the disease progression of SLE via the mediation of phosphatidylserine.

Studies have also identified the Akt/mTOR signalling pathway which is involved in regulating the induction of autophagy and plays an important role in the disease progression of lupus nephritis caused by SLE. The expression *miR-20a* in EVs inhibits the Akt/mTOR pathway. The inhibition of this pathway further increased autophagy markers: Beclin1, LC-II/LC3-1, and p62. The increased autophagy markers reduce the accumulation of IgG and C3 in the glomerular mesangial, thus protecting the podocytes, and can be confirmed by the increased levels of podocin and nephrin [48].

Other studies that were excluded in the PRISMA framework recorded a potential therapeutic effect of EVs on the cGAS-STING pathway as there is a co-relevance of SLE and the production of type 1 IFN through the pathway [58]. Moreover, the use of *miR-21* in EVs as signalling molecules to activate the TLR8 pathway has provided a breakthrough that this pathway has an effect on the disease progression of SLE, and inhibiting it will result in reducing the disease amelioration [59].

## 9. Prospects for Future Research

### 9.1. Standardisation of EVs

The studies have recorded the potential of EVs and their immunomodulatory functions in slowing down the disease progression of SLE. Although EVs have the potential to revolutionise therapeutics for SLE, the current challenge faced in transitioning EV therapeutics into clinical studies is the standardisation of EVs. Due to their heterogenicity, EVs are yet to be standardised, thus affecting regulatory approval for registering EVs as therapeutics.

Prospects in the standardisation of EVs may include identifying common single specific markers present in the EVs and identifying the specific markers resulting in a homogenous selection. However, the single specific markers on EVs cannot be characterised by flow cytometers due to their limitation in detecting and characterising specific markers on EVs. Developing a detection method/equipment similar to the mechanism of flow cytometry can enable the identification of single specific markers on EVs as well as the specific contents of EVs that can provide a breakthrough in the standardisation of EVs. Apart from specific markers, the prospects also include developing instruments that can measure the concentration of EVs accurately and inventing indicators/markers that can determine the release rate of EVs in the body. The markers can further confirm the efficacy of EVs on improving the disease progression of diseases [60].

Furthermore, developing an assay to detect the potency of EVs can be used to standardise EVs based on potency. The potency assay can vary based on disease as well as the specific functionality of EVs that is assessed. Future studies should also consider including a unique EV potency unit (EVsPU) to quantify and standardise methods of characterisation and isolation of EVs to minimise the heterogenicity of EVs obtained from samples [61].

Determining the EV reference material is an important step in standardising EVs. The reference material acts as a guideline to standardise the EV population and determine the standard minimum level of proteins encapsulated in each vesicle. The reference material should include EV measurements that can be reproduced through identified instruments in laboratories based on a standard representation of EV preparation [60].

Concerning standardisation, future studies may include the isolation of EVs from a standardised cell line that has minimal heterogenicity. For example, induced pluripotent stem cells have the potential to differentiate into MSCs. EVs isolated from these MSCs will be more specific and homogenous compared to EVs derived from various sources of MSCs [62].

### 9.2. Enhancing Immunomodulatory Effects of EVs

Specific expression and inhibition of miRNA have proved its potential in improving the immune regulation in SLE-related inflammatory pathways. Future studies may include different miRNAs present in EVs that influence the inflammatory pathway in patients with SLE. The miRNAs that can be studied in future research are miR-335 and miR-92a, which influence the SOX4 pathway; miR-150 and anti-miRNA-150 on the suppression of T cells and the inhibition of inflammation; and miR-223, which influences the macrophage differentiation into pro-inflammatory and anti-inflammatory phenotypes.

Further studies can investigate the effect of EVs on different inflammatory signalling pathways, such as JAK-STAT and NF-κB. JAK-STAT is considered one of the most important inflammatory pathways that is involved in the regulation of inflammation in immune cells via signal transduction. Inhibiting the trigger of this pathway using EVs can downregulate the disease progression of SLE.

EVs engineered with miRNAs and tsRNAs are a significant advancement in improving the therapeutic effects of EVs. However, it is a challenge to ensure the effective delivery of these EVs at target sites. Targeted therapy using modified targeting peptides engineered on the surface of the EVs can achieve effective target delivery of EVs. The targeted therapy is essential in patients with complications related to SLE, such as lupus nephritis that affects kidney functions and diffuse alveolar haemorrhage (DHA) that affects the lung tissue. This prospect can ensure the effective delivery of EVs to the lungs and kidneys to ameliorate the disease progression of SLE.

To further enhance the immunomodulatory effects of EVs, clustered regularly interspaced short palindromic repeats (CRISPR/Cas19) can be used to genetically edit mesenchymal stem cells to increase the differentiation of naïve T cells into Treg cells [63]. Treg cells are involved in the suppression of immune response and are a key player in enhancing the immunomodulation effects on the disease progression of SLE. The increased differentiation into Treg cells can produce EVs with higher immunomodulation effects.

Future studies may include the potential of CRISPR/Cas19 in genetically modifying MSCs to allow the differentiation of MSCs into regulatory immune cells—Treg cells. The immune cells will carry higher immunomodulation effects, and EVs derived from the immune cells will specifically express immunomodulatory effects to suppress the overactivation of the immune response in patients with SLE.

Apart from CRISPR/Cas19, prospects may include the integration of engineering in biology by adapting electromagnetic fields (EMFs), which are the combination of electrical and magnetic fields that produce nonionised and nonthermal waves. EMFs have the potential to be used on Treg cells and M2 macrophages to increase the secretion of anti-inflammatory cytokines via the use of small electric currents and low-frequency EMFs. The enhanced secretion of anti-inflammatory cytokines has the potential to manage and reduce the disease progression of SLE.

## 10. Conclusions

The noteworthy evidence related to the immunomodulatory functions of EVs derived from MSCs in managing the disease progression of SLE has been studied in this review paper. EVs showcase promising characteristics as a therapeutic agent concerning the regulation of T and Treg cells, inhibition of B cells, M2 macrophage polarisation, expression of autophagy markers, and depolarisation of M1 macrophages. The identification of immunomodulatory-related miRNAs and mRNAs in EVs and their specific overexpression and inhibition can further enhance the immunomodulatory effects of EVs in managing SLE. EVs and the specific miRNAs and mRNAs have proven to have a positive impact on inflammatory-related pathways. Further studies should include different miRNAs and the mechanism of action in targeting the inflammatory-related pathway to further improve the therapeutic effects of EVs. Apart from that, further studies should include CRISPR and its potential to further improve the immunomodulation effects of EVs derived from MSCs. Integration of electromagnetic fields and small electric signals can further improve the immunomodulatory functions of EVs derived from MSCs with minimal genetic modification that may or may not have adverse effects on the EVs. EVs hold endless possibilities in the field of therapeutics. A proper understanding of its mechanism will further enhance its potential in the therapeutics of SLE and other autoimmune-related diseases such as rheumatoid arthritis (RA) and juvenile idiopathic arthritis (JIA).

## Figures and Tables

**Table 1:** Description of included studies.

Author	EVs Type/miRNA Studied/Control	Disease Model	Modifications	EV Characterisation (Method) and Tracking	Pathway of Action	
Chen et al. [43]	miRNA overexpression Exosomes hUC-MSCs NOTCH1 overexpression or inhibition Control: PBS	C57BL/6J mice were induced with 0.5 mL pristane.	miR-146a-5p antagonist and agonist transfected to show inhibition/ overexpression	TEM Western blot assay to detect CD9 and CD36 NTA	**miRNA expression:** Antagomir miR-146a-5p shows lung injuries. Higher expression of NOTCH1, IL-1β, and iNOS. miR-146a-5p lowers levels of IL-10 and TGF-β. Exosomes inhibit NOTCH 1 expression by promoting the polarisation of M2 macrophage via miR-146a-5p.	**Control** Antagomir NC (exo). No significant difference in miR-146a-5p, NOTCH1, IL-1β, iNOS, CD206, Arg-1, and IL-10 expression levels.
			No Modifications		**EVs:** Bleeding and inflammation in the lung tissues were alleviated post-treatment. Decreased levels of NOTCH1, IL-1β, and iNOS levels while increased levels of CD206, Arginase-1, and IL-10.	**Control** PBS Healthy Group (No evident changes).
			Overexpression modified with pcDNA3.1—NOTCH1 or pcDNA2.1 Inhibition—short hairpin (sh) RNA lentiviral vectors		**NOTCH 1 Overexpression:** Aggravated bleeding and inflammation. NOTCH1, IL-1β, and iNOS levels increased while CD206, Arginase-1, and IL-10 decreased. IL-6 and TNF-α concentrations increased while IL-10 and TGF-β decreased. **NOTCH 1 Inhibition:** Degree of injuries was reduced, and M2 macrophage polarisation was accelerated by sh-NOTCH1. Reducing NOTCH1, IL-1β, and iNOS. Enhanced CD206, Arginase-1, and IL-10 anti-inflammatory markers. Reduced expression of IL-6, TNF-α, and increased IL-10, or TGF-β.	**Control** (Agomir NC, Con) No difference in expression of miR-146a-5p, NOTCH1, IL-1 β, iNOS, CD206, Arg-1, and IL-10.
Dou et al. [49]	tsRNA expression and Inhibition Human BMMSCs Exosomes Control: PBS	In vitro TPH-1 cell line + PMA to induce macrophage differentiation. (Mononuclear macrophage line)	tsRNA-21109 inhibition and expression	Hollow spherical microvesicle morphology Expression of CD36 Size: (100–200 nm) TEM	**tsRNA expression:** Reduced expression of tsRNA-21109 can be seen in patients with SLE. tsRNA-21109 can inhibit the M1 macrophage polarisation through Rap1, Ras, Hippo, Wnt, MAPK, and TGF-beta signalling pathways. **Inhibition of tsRNA-21109:** This leads to increased TNF-α and IL-1β, CD80, NOS2, and MCP-1 markers. Decreased in M2 markers CD206, MRC-2, and Arg-1.	**Control** MSCs-exo showed lower levels of CD80/ARG-1 compared to tsRNA-21109 inhibition.
			Exosomes derived from MSCs		**BMMSC EVs:** Decrease in M1/M2 polarisation where expression of CD80, NOS2, and MCP-1 were decreased while CD206, MRC-2, and ARG1 increased. Reduction in TNF-α and IL-1β. Changed the TRF expression of M1 macrophages.	**Control:** Untreated macrophage culture showed expression of CD80, NOS2, and MCP-1 while reduction in CD206, MRC-2, and ARG-1.
Sonoda et al. [50]	RNA and miRNA expression (SHED-EVs) SHED-EVs on BMMSC EVs Control: PBS	In vitro and In vivo Immunocompromised NOD-SCID mice BM-MSCs derived from NOD-SCID mice.	Small interfering RNA for Tert (siRNATert) and RAB27A (siRNARAB27A)	Surface Ag expression was analysed using an ExoAB Ab kit and R-PE-conjugated anti-rabbit IgG AB Particle size measured with NTA (69–478 nm)	**RNA and siRNA on SHED EVs and effect on BMMSCs:** MIR346 binds to a region in the TERTmRNA to upregulate TERT expression. Improves the functions of hematopoietic niche formation and immune regulation in recipient BMMSCs through the epigenetically regulated TERT telomerase activity pathway.	**Control** siRNACont (silencing RNA) Expression of Sca-1+, c-kit+, and CD45+ cells.
			No Modifications		**SHED EVs:** Reduced the peripheral autoantibody levels, renal functions, and levels of CD4+ IL-17+ IFN-gamma and increased CD4+ CD25+ Foxp3+ in the PBMCs of the mice.	**Control** PBS solution Nontransplanted control (lpr-BMMSCs). Control wild type (B6-BMMSCs). Did not recover the TERT expression and telomerase activity. Reduced levels of Sca-1+, c-kit+, and CD45+ cells.
			No Modifications		**SHED EVs on BMMSC EVs:** Reduced CD4+ IL-17+ IFN-gamma cells and increased levels of CD4+ CD25+ Foxp3+ (Treg cells) and Annexin-V+ 7AAD+ cells. Reduced disease progression of SLE in mice.	
Tu et al. [44]	miR-19b expression UC-MSCs Exosomes Control: Healthy control group/ Negative control	In vitro model CD4+ cells of PBMC cells	miR-19b derived UC-MSCs that are transduced into CD4+ T cells via exosomes.	Rotund or oval-shaped exosomes NTA Size of exosomes: 132.5 (±) 37.4 nm Expressed CD63 and TSG101	**miR-19b expression:** Lowered the levels of TNF, IL-6, and IL-17 and increased levels of IL-10 and TGF-beta. miR-19b inhibited the endogenous expression of KLF13 in T cells. Exosomes derived from UC-MSCs promote the expression of T cells in expressing miR-19b to inhibit the level of KLF13 and improve Th17/Treg cells regulation.	**Control** miR-19b NC miR-19b higher in PBMC of normal controls. Lower KLF13, TNF-α, IL-6, IL-17, and increased IL-10 and TGF- β.
Wei et al. [48]	miR-20a overexpression Adipose tissue-derived stem cells Exosomes Control: PBS	B6.MRL/lpr mice C57BL/6 mice	ADSCs transfected with miR-20a to produce miR-20a-ADSCs-derived exosomes.	CD63 marker was used to identify exosomes. None	**miR-20a overexpression:** Anti-dsDNA antibody, urine protein, and serum creatinine levels are the lowest in miR-20a-ADSC. Inhibited the Akt/mTOR pathway (LN pathogenesis). Higher autophagy markers of Beclin 1, LC3-II/LC3-I, and p62. Increased podocin and nephrin and higher autophagosomes. Reduced podocyte damage through autophagy. Fewer histopathologic abnormalities reduced C3 and IgG deposits and reduced nephritis scores.	**Control** Empty lentiviral vector (NC) Normal levels of Anti-dsDNA level and serum creatine. Lower C3 deposits in glomeruli, higher levels of Beclin 1 and LC3-II/LC3-1 and p62. Higher podocin and nephrin expression. Higher autophagosomes.
Zhang et al. [51]	miR-16 and miR-21 overexpression and inhibition BMMSCs Exosomes Control: PBS	In Vitro C57BL/6 mice were induced with pristane oil to obtain macrophage from the kidney. In vivo macrophages overexpression of miR-16 and miR-21	BM-MSCs transduced with lenti-anti-miR-16 and lenti-anti-miR-21 (Exo/Anti-miRNAs)	Size of exosomes: 40 nm Exosome markers were detected using the immunoblot analysis (Alix and TSG-101) ExoGlow-Protein EV labelling kit (green, fluorescent dye)	**miR-16 and miR-21 overexpression and inhibition:** Reduced proinflammatory cytokines and increased anti-inflammatory cytokines. Reduction in iNOS, PDCD4, and PTEN levels. High expression of CCL20 (ligand on t cells). Higher expression of CD206, Arg-1, B7H4, and CD138 but lower expression of CD86 and iNOS. Macrophages ingested more apoptotic cells. Macrophage polarisation is contributed by the exosomes carrying miR-16 and miR-21 via targeting PDCD4 and PTEN. Increased efferocytosis of apoptotic cells. More production of Treg cells.	**Control** Macrophages transduced with Lent/ZIPmiR-Cont (ZIPmiR-Cont) Increased levels of miR-16 and miR-21 were elevated, and levels of iNOS, PDCD4, and PTEN decreased. Arg-1, CD206, B7H4 and CD138 markers increased.
			No modifications		**BMMSC EVs:** Immune cell infiltration in the renal interstitium and mesangial expansion were significantly reduced. Lessened immune deposits (IgG, IgM, C3, and C1q). Reduced T cell infiltration. High levels of CD206 showing macrophage M2 polarisation. Reduced CD86 and increased CD206. Downregulated iNOS and upregulated Arg-1. Pro-inflammatory cytokines IFN-γ, IL-1β, IL-6, IL-12, and GM-CSF were reduced and anti-inflammatory cytokines IL-10, TGF-β, and M-CSF were increased. Reduced levels of ROS show a shift in macrophage polarisation. T cells differentiate into Treg cells.	
Zhao et al. [45]	miR-155 Inhibition Exosomes derived from (hucMSCs) Control: Healthy patients	In vitro PBMCs of SLE patients	No Modifications	Vesicles with a sphere shape in a size range of 130 nm NTA to track the exosomes. TEM to determine the morphology. Surface markers CD63 and CD81	**miR-155 inhibition:** Inhibition of miR155- in B cells increases the SHIP-1 levels, reducing the activation of B cells. SHIP-1 activity promotes the apoptosis of B cells besides inhibiting its proliferation and overactivation. Inhibition of the ERK/SHIP-1 pathway can reduce inflammation. Inhibition of miR155 reduced IL-6 and TNF-α.	**Control** Lower expression of miR-155. Decreased levels of SHIP-1. Antagomir NC recorded lower levels of B cell apoptosis. Expressed IL-6 and TNF-α. p-ERK activation in B cells was not inhibited
			No Modifications		**EVs from hucMSCs:** Reduced B cell hyperactivation. Reduced cytokine levels of IL-16, IL-10, INF-γ, IL-17, 1L-4, and TNF-α. High levels of IL-6- due to paracrine effect from MSCs.	**Control** Does not inhibit B cell proliferation. Does not overactivate B cells. IL-16, IL-10 and TNF-α were expressed. IL-6 is lower in the control group. Lower expression of miR-155.
Chen et al. [46]	hucMSCs-Exo Control: PBS	C57BL/6J mice (SPF) induced by pristine to develop DAH	No Modifications	Round or elliptical shape with an intact capsule Exosome markers CD63, TSG101, and Alix Diameter range: 40–120 nm	**EVs derived from hUC-MSC:** Enhances the polarisation of M2 macrophage to alleviate DAH. Improved phagocytosis of macrophages in DAH. Facilitated the transformation of macrophages from M1 to M2 phenotype. iNOS, IL-6, TNF-α, and IL-1β deceased in M1 macrophage phenotype. Arg1, IL-10, TGF-β, and chi3l3 levels were upregulated in the M2 macrophage phenotype.	**Control** No changes in the F4/80 + CD11b + CD86 + CD206− cells. No changes in IL-6, iNOS, TNF-α, IL-10, and TGF- β levels. No changes in phagocytosis
Sun et al. [47]	hucMSCs-Exo Control: PBS	In vitro and In vivo pTHP-1 macrophages C57BL/6lpr-/-(B6.lpr)	No Modifications	The saucer-like shape of exosomes The diameter of exosomes ranged from 80 nm to 150 nm Markers determined from Western blot: CD9, TSG101, and GAPDH	**EVs derived from hucMSCs (In vitro)** Macrophage proliferation, inflammation, and M1 polarisation were inhibited post-treatment. Reduced expression of IL-1β and TNF-α and increased CD163+ M cells and CD206 + M cells but reduced HLA-DR+ and CD68+ cells. (**In vivo)** Reduced deposition of C3 immune complex. Increased CD14+ CD163+ markers of M2 cells and decreased CD14 + CD11c+ markers of M1 cells. The collagen fibre deposition in the glomeruli was significantly reduced. Increased serum IL-10 and decreased serum TNF-α and IFN-α. Increased CD4+ CD25 + FoxP3+ Treg cells. Alleviated nephritis and lung injury in MRL/lpr mice. Promoted the polarisation of M2 and Treg in MRL/lpr mice and the survival of mice. Reduction in pulmonary septal thickening and fibrosis.	**Control** No expression of IL-1 β and TNF-α. No significant difference in the deposition of C3 in glomeruli. No reduction in pulmonary septal thickening and fibrosis. Expression of CD14+ CD11c+ M1 cell infiltration. Lower CD14+ CD163+ M2 cells compared to EV treatment. No changes in IL-10, TNF-α and IFN-α.
Xie et al. [1]	hucMSCs-Exo Control: PBS	In vitro Splenic mononuclear cells of MRL/lpr mice	No modifications	Size of exosomes: 139.1 nm diameter TEM and NTA	**EVs derived from hucMSCs:** CD4+ T cells undergone inhibition in splenic mononuclear cells of MRL/lpr mice. Promoted Th17 cell differentiation. Increased the cytokine concentration of IL-17. The cytokine concentration of TGF -was increased.	**Control** Higher CD4+ T cells Lower Th17 cells Reduced concentration of IFNγ Lower levels of IL-4.
Wang et al. [52]	(BMMSCs) derived from C57b16. Apoptotic vesicles (ApoVs) derived from apoptotic BMMSCs.	In Vitro and In Vivo MRL/Ipr mice Apoptosis induced with staurosporine (STS)	No modifications	Spherical shape apoVs size = (100–350 nm) Apoptosis marker PtdSer/PS to validate the apoVs	**apoVs derived from BMMSCs (in vivo):** Decreased T cells in lymphoid tissue. Increased CD4+ T effector cells. Decreased CD4+ T naïve cells. Reduction in IFNγ+ and CD4+ T cells. Increased Foxp3+ CD4 + T cells effector. Reduction in lymphoproliferation. Reduced levels of anti-dsDNA and IgG. **(In vitro)** Reduced expansion of Th1 (IFNγ + CD4+), Th17 (IL-17A + CD4+) and Th2 (IL-4 + CD4+). Reduced levels of cytokines, including IFNγ, IL-17A, and IL-10. Foxp3+ and CD4+ T cells remained the same. Suppressed CD4+ T cells and IL-2 levels. ApoEVs diminished the phosphorylation of the TCR signalling pathway.	**Control** Non-Apoptotic control Higher Th17 cells. Severe arthritis.

BMMSCS, bone marrow mesenchymal stromal cells; SHED, stem cells from human exfoliated deciduous teeth; hucMSCs, human umbilical cord mesenchymal stromal cells; PBS, phosphate buffer solution; TEM, transmission electron microscopy; NTA, nanoparticle tracking analyser; STS, staurosporine; PtdSer, phosphatidylserine UC-MSCs, umbilical cord mesenchymal stromal cells; PBMCs, peripheral blood mononuclear cells; DAH, diffuse alveolar haemorrhage; pTHP-1, human leukaemia monocytic cell line; TCR, T cell receptor; Exo, exosomes; EVs, extracellular vesicles.

**Table 2:** Simplified description of included studies.

Study	Disease Model	Source of EVs	Range of Dose	Experimental Groups	Outcome
[43]	DAH mouse model induced with pristane.	hUC-MSCs	100 μL of 0.2 mg/mL EVs via intravenous injection through the tail vein every 2 days for 14 days.	*n* = 90 female rats 5 groups Healthy Induced with Pristane Exosome treatment NOTCH 1 expression Knockdown of NOTCH1	Promote polarisation of macrophages into M2 phenotype. Increases anti-inflammatory cytokines. Alleviation of bleeding and inflammation in the lungs.
[49]	In-Vitro TPH-1 cell line+PMA to induce macrophage polarisation and IFN γ+LPS to induce M1 phenotype.	BM-MSCs	None	None	Decrease in M1 polarisation and increase the polarisation into M2 phenotype. Reduction pro-inflammatory cytokines and increased levels of anti-inflammatory cytokines.
[50]	In Vivo Immunocompromised NOD-SCID mice In-Vitro BM-MSCs derived from NOD-SCID mice.	SHED	100 μg of SHED EVs in 100 mL of PBS solution once in 4 weeks.	3 groups SHED transplanted MRL/Ipr Non-transplanted control MRL/Ipr Control Wild Type C57/BL6	Increased anti-inflammatory cytokines. Reduced disease progression of SLE. Recovered immune condition and renal functions.
[44]	In-Vitro Model CD4+ cells of PBMC cells.	UC-MSCs	None	None	Down-regulation of pro-inflammatory cytokines and up-regulation of anti-inflammatory cytokines. Regulation of Treg and T helper cells.
[48]	B6.MRL/Ipr Mice and C57BL/6 mice.	ADSC	2 × 10^5^ cells per 10 g animal weight in 150 μL for 14 days	4 groups Lupus group (*n* = 10) ADSC group (*n* = 10) Control group (*n* = 10) miR-20a group (*n* = 10)	Reduction of disease severity and delayed lupus nephritis progression. Activation of autophagy and reduction of podocyte damage
[51]	In-Vitro Macrophage obtained from kidney of pristane induced mice.	BM-MSCs	None	None	Increased differentiation of T cells into Treg cells. Increased expression of anti-inflammatory markers. Reduction of lupus nephritis.
[45]	In-Vitro PBMC of SLE patients.	UC-MSCs	None	None	Increased immunomodulatory effects of B cells. Promotion of B cell apoptosis, prevention of B cell proliferation and activation. Regulation of the autoimmune reaction in PBMC of SLE patients.
[46]	DAH mouse model induced with pristane	hUC-MSCs	100 μL of 0.2 mg/mL EVs via intravenous injection through the tail vein every 2 days for 14 days	*n* = 40 4 groups DAH DAH+exo DAH+ methylprednisolone Control	Promoted polarisation of macrophages into M2 phenotype. Increased anti-inflammatory cytokines. Improved phagocytosis of macrophages.
[47]	In-vitro pTHP-1 macrophages In-vivo C57BL/6lpr-/- (B6.lpr)	hUC-MSCs	Single dose of EVs of 200 μg protein concentration.	*n* = 18 3 groups Control group. Exosomes group C57BL/6lpr-/- (B6.lpr)- lupus model	Inhibited the proliferation of macrophages. Inhibited inflammation and M1 polarisation. Alleviated nephritis and lung injuries in mice. Promote polarisation of M2 and Treg in mice. Increased survival of mice.
[1]	In-Vitro Splenic Mononuclear Cells	hUC-MSCs	None	None	Promotion of the differentiation of Th17 cells. Regulation of immune cells and cytokine levels.
[52]	In Vitro & In Vivo MRL/Ipr mice	ApoVs BM-MSCs	Weekly administration for 4 weeks	3 groups MRL/MpJ MRL/Ipr MRL/Ipr treated with apoVs	Promotion of immunomodulatory effects on T cells. Amelioration of inflammation.

BMMSCS, bone marrow mesenchymal stromal cells; SHED, stem cells from human exfoliated deciduous teeth; hucMSCs, human umbilical cord mesenchymal stromal cells; PBS, phosphate buffer solution; ADSC, adipose-derived stem cells; STS, staurosporine; PtdSer, phosphatidylserine; UC-MSCs, umbilical cord mesenchymal stromal cells; PBMCs, peripheral blood mononuclear cells; DAH, diffuse alveolar haemorrhage; pTHP-1, human leukaemia monocytic cell line; Exo, exosomes; EVs, extracellular vesicles.

## Data Availability

Data will be made available on request.

## Supplementary Materials

The following supporting information can be downloaded at: https://www.mdpi.com/article/10.3390/ijms25042444/s1.
