# Supplementary figures and images for: Potential Therapeutic Application and Mechanism of Action of Stem Cell-Derived Extracellular Vesicles (EVs) in Systemic Lupus Erythematosus (SLE)
Source: Int J Mol Sci. 2024 Feb 19;25(4):2444. doi: 10.3390/ijms25042444 (PMC10889333; doi:10.3390/ijms25042444)

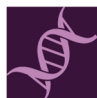

---

**Supplementary File S1** Schematic Representation of the Included Studies.

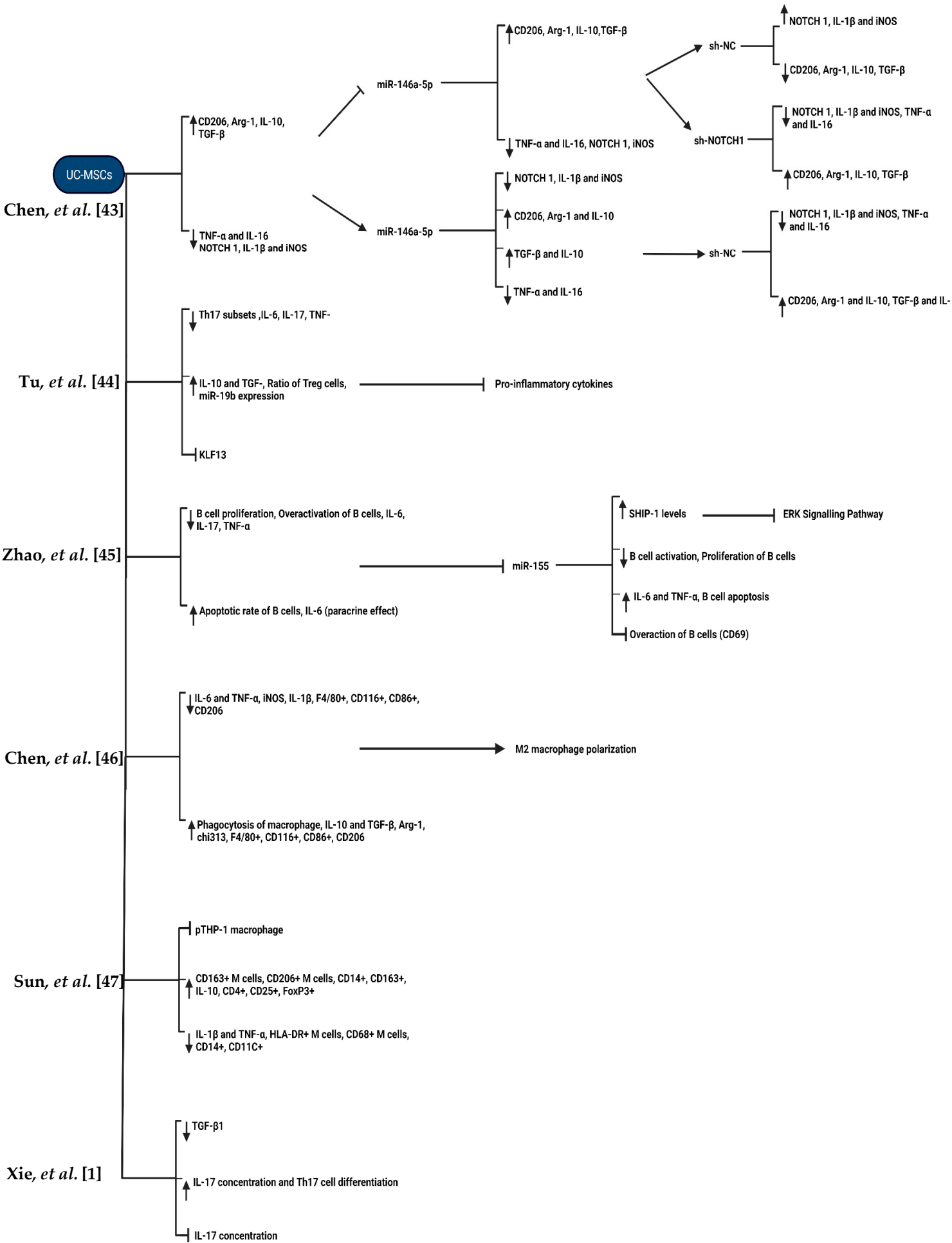

(continued)

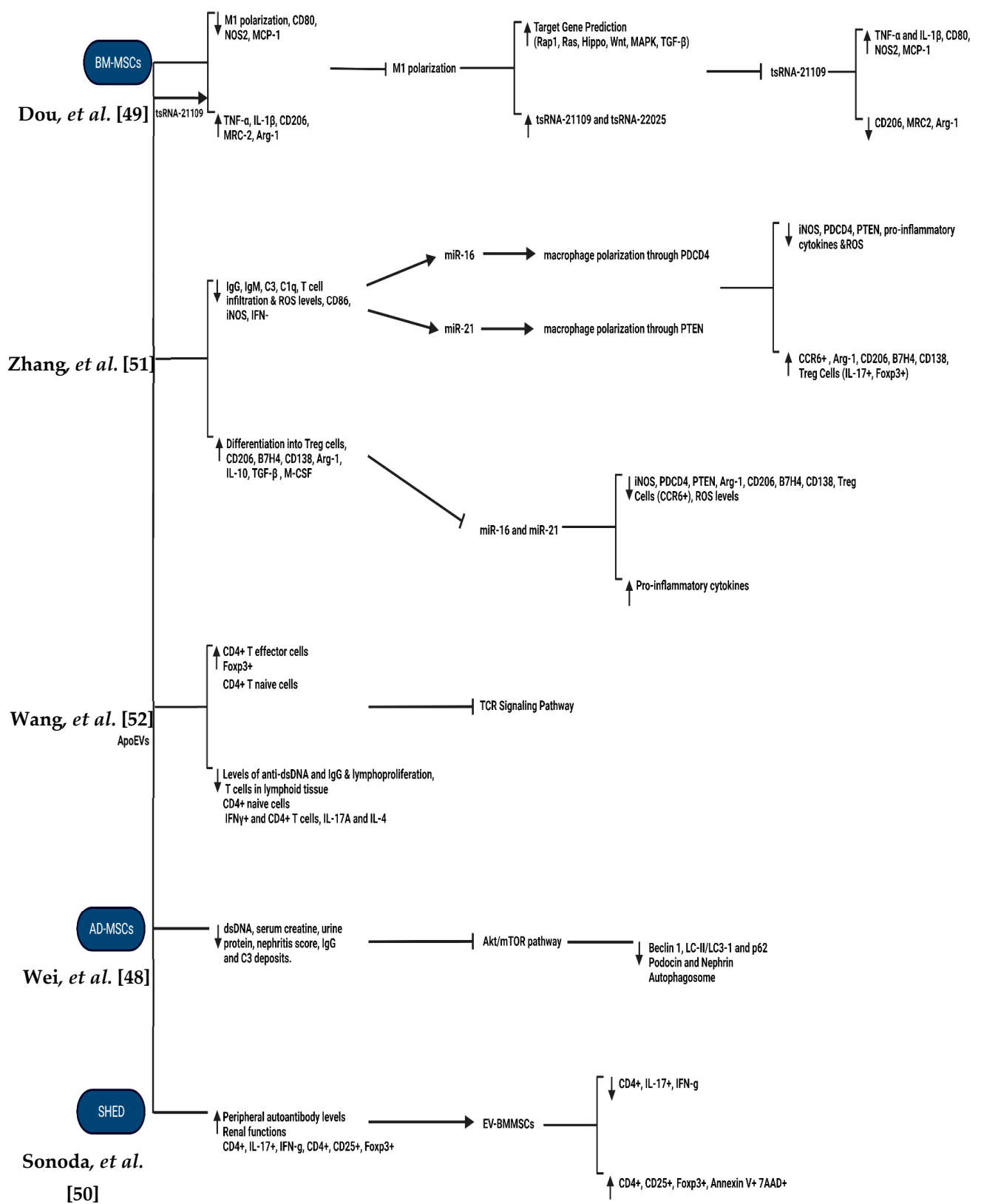

Supplement: Supplementary file 1 [file ijms-25-02444-s001.zip › ijms-2843576-supplementary.pdf]
